# (*E*)-4-Hy­droxy-*N*′-(2-hy­droxy-3,5-diiodo­benzyl­idene)-3-meth­oxy­benzohydrazide methanol monosolvate

**DOI:** 10.1107/S1600536812004552

**Published:** 2012-02-10

**Authors:** Xiao-Yan Li

**Affiliations:** a Zibo Vocational Institute, Zibo 255314, People’s Republic of China

## Abstract

In the title compound, C_15_H_12_I_2_N_2_O_4_·CH_3_OH, the hydrazone mol­ecule exists in an *E* conformation with respect to the C=N bond. The dihedral angle between the rings is 11.9 (2)°. There is one intra­molecular O—H⋯N hydrogen bond in the hydrazone mol­ecule. In the crystal, the hydrazone and methanol mol­ecules are linked through O—H⋯O and N—H⋯O hydrogen bonds and C—H⋯O inter­actions to form two-dimensional networks lying parallel to (001).

## Related literature

For the syntheses and crystal structures of hydrazone compounds, see: Hashemian *et al.* (2011 ▶); Lei (2011 ▶); Shalash *et al.* (2010 ▶). For the crystal structures of similar compounds, reported recently by the author, see: Li (2011*a* ▶,*b* ▶).
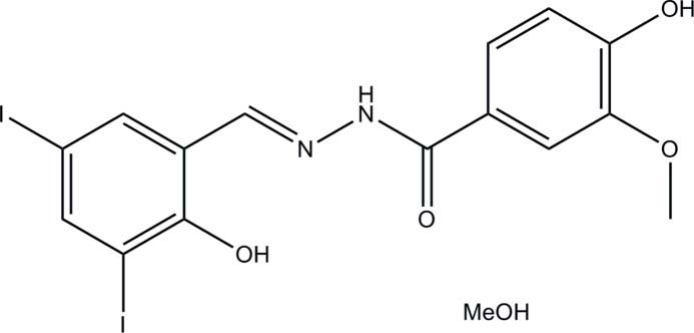


## Experimental

### 

#### Crystal data

C_15_H_12_I_2_N_2_O_4_·CH_4_O
*M* *_r_* = 570.11
Orthorhombic, 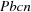

*a* = 19.467 (3) Å
*b* = 12.655 (2) Å
*c* = 16.138 (2) Å
*V* = 3975.5 (11) Å^3^
*Z* = 8
Mo *K*α radiation
μ = 3.19 mm^−1^
*T* = 298 K
0.23 × 0.20 × 0.20 mm

#### Data collection

Bruker SMART CCD area-detector diffractometer
Absorption correction: multi-scan (*SADABS*; Sheldrick, 1996 ▶) *T*_min_ = 0.527, *T*_max_ = 0.568
22354 measured reflections
4315 independent reflections
3198 reflections with *I* > 2σ(*I*)
*R*_int_ = 0.040

#### Refinement

*R*[*F* ^2^ > 2σ(*F* ^2^)] = 0.039
*wR*(*F* ^2^) = 0.091
*S* = 1.02
4315 reflections
239 parameters
3 restraints
H atoms treated by a mixture of independent and constrained refinement
Δρ_max_ = 1.12 e Å^−3^
Δρ_min_ = −1.35 e Å^−3^

### 

Data collection: *SMART* (Bruker, 1998 ▶); cell refinement: *SAINT* (Bruker, 1998 ▶); data reduction: *SAINT*; program(s) used to solve structure: *SHELXS97* (Sheldrick, 2008 ▶); program(s) used to refine structure: *SHELXL97* (Sheldrick, 2008 ▶); molecular graphics: *SHELXTL* (Sheldrick, 2008 ▶); software used to prepare material for publication: *SHELXTL*.

## Supplementary Material

Crystal structure: contains datablock(s) global, I. DOI: 10.1107/S1600536812004552/su2374sup1.cif

Structure factors: contains datablock(s) I. DOI: 10.1107/S1600536812004552/su2374Isup2.hkl

Supplementary material file. DOI: 10.1107/S1600536812004552/su2374Isup3.cml

Additional supplementary materials:  crystallographic information; 3D view; checkCIF report

## Figures and Tables

**Table 1:** Hydrogen-bond geometry (Å, °)

*D*—H⋯*A*	*D*—H	H⋯*A*	*D*⋯*A*	*D*—H⋯*A*
O1—H1⋯N1	0.82	1.89	2.614 (4)	147
O5—H5⋯O2	0.85 (3)	1.87 (2)	2.698 (4)	165 (5)
N2—H2⋯O3^i^	0.91 (4)	2.17 (5)	3.024 (4)	157 (3)
O3—H3⋯O5^ii^	0.85 (5)	1.80 (4)	2.643 (4)	170 (4)
C14—H14⋯O3^i^	0.93	2.55	3.442 (5)	162
C16—H16*A*⋯O1	0.96	2.51	3.267 (7)	135

Symmetry codes: (i) 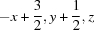; (ii) 
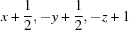
.

**Fig. 1.**
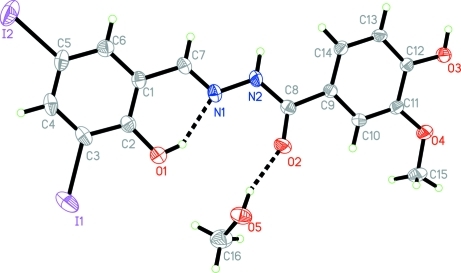
The molecular structure of the title compound, showing the atom labelling scheme. The displacement ellipsoids are drawn at the 30% probability level. The intramolecular O–H···N hydrogen bond, and the O-H···O hydrogen bond linking the hydrazone and methanol molecules are indicated by dashed lines (see Table 1 for details).

**Fig. 2.**
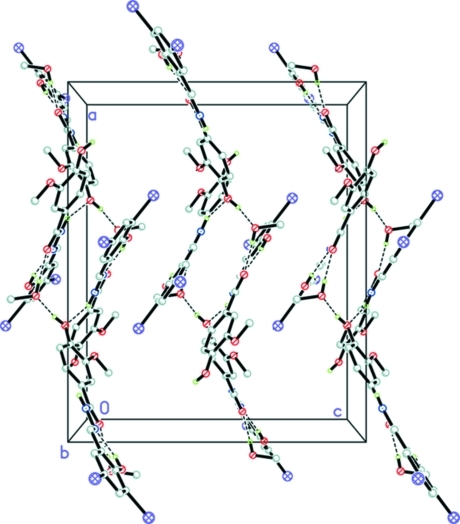
Crystal packing of the title compound, viewed along the b axis. Hydrogen bonds are indicated by dashed lines (see Table 1 for details). The C-bound H-atoms have been omitted for clarity.

## supplementary crystallographic information

### Comment

In recent years, hydrazone compounds have attracted much attention due to their syntheses and crystal structures (Hashemian *et al.*, 2011; Lei, 2011; Shalash *et al.*, 2010). As a continuation of our work on such compounds (Li, 2011*a*,b), the author reports herein on the crystal structure of the new title hydrazone compound.

The title compound (Fig. 1), contains a *N*'-(2-hydroxy-3,5-diiodobenzylidene)-4-hydroxy-3-methoxybenzohydrazide molecule and a methanol solvent molecule. The hydrazone molecule exists in a *trans* or *E* conformation with respect to the C7═N1 bond. The dihedral angle between the (C1–C6) and (C9–C14) benzene rings of the hydrazone molecule is 11.9 (2)°. There is one O–H···N intramolecular hydrogen bond in the hydrazone molecule (Table 1).

In the crystal, the hydrazone and methanol molecules are linked through O–H···O and N–H···O hydrogen bonds and C-H···O interactions (Table 1), to form a two-dimensional network lieing parallel to the ab plane (Fig. 2).

### Experimental

A mixture of 2-hydroxy-3,5-diiodobenzaldehyde (0.374 g, 1 mmol) and 4-hydroxy-3-methoxybenzohydrazide (0.182 g, 1 mmol) in 30 ml of ethanol containing few drops of acetic acid was refluxed for about 1 h. On cooling to room temperature, a solid precipitate was formed. The solid was filtered and then recrystallized from methanol. Colourless crystals, suitable for X-ray diffraction analysis, were obtained by slow evaporation of a solution of the title compound in methanol.

### Refinement

Hydrogen atoms H2, H3, and H5 were located in a difference Fourier map and were freely refined. The remaining H-atoms were positioned geometrically and refined using a riding model: O–H = 0.82 Å, C–H = 0.93 and 0.96 Å for CH and CH_3_ H atoms, respectively, with U_iso_(H) = k × U_eq_(O,C), where k = 1.5 for OH and CH_3_ H-atoms, and k = 1.2 for all other H-atoms.

### Crystal data

**Table:**

C_15_H_12_I_2_N_2_O_4_·CH_4_O	*F*(000) = 2176
*M**_r_* = 570.11	*D*_x_ = 1.905 Mg m^−^^3^
Orthorhombic, *P**b**c**n*	Mo *K*α radiation, λ = 0.71073 Å
Hall symbol: -P 2n 2ab	Cell parameters from 6406 reflections
*a* = 19.467 (3) Å	θ = 2.3–26.0°
*b* = 12.655 (2) Å	µ = 3.19 mm^−^^1^
*c* = 16.138 (2) Å	*T* = 298 K
*V* = 3975.5 (11) Å^3^	Block, colourless
*Z* = 8	0.23 × 0.20 × 0.20 mm

### Data collection

**Table:**

Bruker SMART CCD area-detector diffractometer	4315 independent reflections
Radiation source: fine-focus sealed tube	3198 reflections with *I* > 2σ(*I*)
Graphite monochromator	*R*_int_ = 0.040
ω scans	θ_max_ = 27.0°, θ_min_ = 1.9°
Absorption correction: multi-scan (*SADABS*; Sheldrick, 1996)	*h* = −24→24
*T*_min_ = 0.527, *T*_max_ = 0.568	*k* = −15→15
22354 measured reflections	*l* = −20→17

### Refinement

**Table:**

Refinement on *F*^2^	Secondary atom site location: difference Fourier map
Least-squares matrix: full	Hydrogen site location: inferred from neighbouring sites
*R*[*F*^2^ > 2σ(*F*^2^)] = 0.039	H atoms treated by a mixture of independent and constrained refinement
*wR*(*F*^2^) = 0.091	*w* = 1/[σ^2^(*F*_o_^2^) + (0.027*P*)^2^ + 9.4128*P*] where *P* = (*F*_o_^2^ + 2*F*_c_^2^)/3
*S* = 1.02	(Δ/σ)_max_ < 0.001
4315 reflections	Δρ_max_ = 1.12 e Å^−^^3^
239 parameters	Δρ_min_ = −1.35 e Å^−^^3^
3 restraints	Extinction correction: *SHELXL97* (Sheldrick, 2008), Fc^*^=kFc[1+0.001xFc^2^λ^3^/sin(2θ)]^-1/4^
Primary atom site location: structure-invariant direct methods	Extinction coefficient: 0.00222 (10)

### Special details

**Table:**

Geometry. Bond distances, angles etc. have been calculated using the rounded fractional coordinates. All su's are estimated from the variances of the (full) variance-covariance matrix. The cell esds are taken into account in the estimation of distances, angles and torsion angles
Refinement. Refinement of *F*^2^ against ALL reflections. The weighted *R*-factor *wR* and goodness of fit *S* are based on *F*^2^, conventional *R*-factors *R* are based on *F*, with *F* set to zero for negative *F*^2^. The threshold expression of *F*^2^ > σ(*F*^2^) is used only for calculating *R*-factors(gt) *etc*. and is not relevant to the choice of reflections for refinement. *R*-factors based on *F*^2^ are statistically about twice as large as those based on *F*, and *R*- factors based on ALL data will be even larger.

### Fractional atomic coordinates and isotropic or equivalent isotropic displacement parameters (Å^2^)

**Table:**

	*x*	*y*	*z*	*U*_iso_*/*U*_eq_	
I1	0.30599 (2)	0.65273 (4)	0.22384 (2)	0.0780 (2)	
I2	0.44485 (3)	1.03076 (3)	0.37750 (3)	0.0875 (2)	
O1	0.42798 (16)	0.5423 (2)	0.3180 (2)	0.0600 (11)	
O2	0.53658 (14)	0.3091 (2)	0.40531 (19)	0.0511 (10)	
O3	0.81379 (14)	0.1047 (2)	0.53841 (19)	0.0469 (10)	
O4	0.72081 (16)	0.0227 (2)	0.4401 (2)	0.0579 (11)	
N1	0.53819 (16)	0.5213 (3)	0.4091 (2)	0.0435 (11)	
N2	0.59137 (17)	0.4611 (3)	0.4418 (2)	0.0430 (11)	
C1	0.48852 (19)	0.6898 (3)	0.3790 (2)	0.0400 (12)	
C2	0.4343 (2)	0.6470 (3)	0.3315 (3)	0.0430 (12)	
C3	0.3854 (2)	0.7157 (4)	0.2971 (3)	0.0480 (14)	
C4	0.3886 (2)	0.8237 (4)	0.3112 (3)	0.0550 (16)	
C5	0.4420 (2)	0.8658 (3)	0.3584 (3)	0.0527 (16)	
C6	0.4916 (2)	0.8002 (3)	0.3918 (3)	0.0480 (12)	
C7	0.5422 (2)	0.6220 (3)	0.4159 (3)	0.0427 (12)	
C8	0.58719 (19)	0.3537 (3)	0.4359 (2)	0.0378 (11)	
C9	0.64765 (18)	0.2924 (3)	0.4669 (2)	0.0337 (11)	
C10	0.65308 (19)	0.1866 (3)	0.4410 (2)	0.0393 (12)	
C11	0.70893 (19)	0.1254 (3)	0.4644 (2)	0.0378 (11)	
C12	0.76007 (18)	0.1695 (3)	0.5169 (2)	0.0349 (11)	
C13	0.75389 (19)	0.2734 (3)	0.5434 (2)	0.0382 (11)	
C14	0.69824 (19)	0.3345 (3)	0.5188 (2)	0.0383 (11)	
C15	0.6690 (3)	−0.0275 (4)	0.3916 (4)	0.074 (2)	
O5	0.40228 (15)	0.2948 (3)	0.3656 (2)	0.0545 (10)	
C16	0.3918 (3)	0.2952 (5)	0.2788 (3)	0.072 (2)	
H1	0.45980	0.51100	0.34060	0.0900*	
H2	0.6291 (17)	0.493 (4)	0.463 (3)	0.0800*	
H3	0.839 (2)	0.136 (4)	0.574 (3)	0.0800*	
H4	0.35510	0.86800	0.28920	0.0660*	
H6	0.52730	0.82890	0.42290	0.0570*	
H7	0.57900	0.65240	0.44400	0.0510*	
H10	0.61890	0.15740	0.40790	0.0470*	
H13	0.78720	0.30230	0.57790	0.0460*	
H14	0.69460	0.40400	0.53700	0.0460*	
H15A	0.62660	−0.02830	0.42200	0.1110*	
H15B	0.68270	−0.09870	0.37950	0.1110*	
H15C	0.66280	0.01070	0.34080	0.1110*	
H5	0.4435 (11)	0.311 (4)	0.376 (3)	0.0800*	
H16A	0.39370	0.36660	0.25860	0.1080*	
H16B	0.34770	0.26540	0.26630	0.1080*	
H16C	0.42710	0.25410	0.25240	0.1080*	

### Atomic displacement parameters (Å^2^)

**Table:**

	*U*^11^	*U*^22^	*U*^33^	*U*^12^	*U*^13^	*U*^23^
I1	0.0504 (2)	0.1183 (4)	0.0653 (2)	0.0102 (2)	−0.0165 (2)	−0.0126 (2)
I2	0.1299 (4)	0.0430 (2)	0.0895 (3)	0.0164 (2)	0.0026 (3)	0.0088 (2)
O1	0.0545 (19)	0.0484 (18)	0.077 (2)	0.0036 (14)	−0.0186 (17)	−0.0037 (16)
O2	0.0344 (15)	0.0555 (18)	0.0634 (19)	0.0008 (13)	−0.0115 (14)	−0.0053 (15)
O3	0.0377 (16)	0.0410 (15)	0.0620 (19)	0.0103 (12)	−0.0107 (13)	−0.0080 (14)
O4	0.0627 (19)	0.0359 (16)	0.075 (2)	0.0051 (14)	−0.0197 (17)	−0.0150 (15)
N1	0.0366 (17)	0.046 (2)	0.0480 (19)	0.0088 (15)	−0.0006 (15)	0.0069 (16)
N2	0.0347 (17)	0.0403 (19)	0.054 (2)	0.0076 (14)	−0.0071 (16)	0.0050 (16)
C1	0.036 (2)	0.043 (2)	0.041 (2)	0.0050 (17)	0.0046 (17)	0.0060 (18)
C2	0.038 (2)	0.048 (2)	0.043 (2)	0.0048 (18)	0.0014 (17)	0.0007 (18)
C3	0.037 (2)	0.068 (3)	0.039 (2)	0.010 (2)	−0.0003 (17)	0.005 (2)
C4	0.058 (3)	0.059 (3)	0.048 (2)	0.023 (2)	0.001 (2)	0.014 (2)
C5	0.064 (3)	0.040 (2)	0.054 (3)	0.012 (2)	0.007 (2)	0.011 (2)
C6	0.047 (2)	0.047 (2)	0.050 (2)	−0.0020 (19)	0.002 (2)	0.002 (2)
C7	0.034 (2)	0.049 (2)	0.045 (2)	0.0057 (17)	−0.0022 (17)	0.0046 (18)
C8	0.0325 (19)	0.044 (2)	0.037 (2)	0.0020 (17)	0.0008 (16)	−0.0025 (17)
C9	0.0271 (17)	0.0349 (19)	0.039 (2)	−0.0009 (15)	0.0010 (15)	0.0005 (16)
C10	0.037 (2)	0.038 (2)	0.043 (2)	−0.0053 (16)	−0.0061 (17)	−0.0009 (17)
C11	0.039 (2)	0.0323 (19)	0.042 (2)	−0.0013 (15)	0.0000 (17)	−0.0040 (16)
C12	0.0302 (18)	0.0335 (19)	0.041 (2)	0.0017 (15)	0.0013 (15)	−0.0006 (16)
C13	0.0305 (18)	0.038 (2)	0.046 (2)	−0.0033 (16)	−0.0053 (17)	−0.0059 (17)
C14	0.0365 (19)	0.0314 (19)	0.047 (2)	0.0013 (16)	−0.0014 (17)	−0.0025 (17)
C15	0.087 (4)	0.045 (3)	0.090 (4)	−0.004 (3)	−0.028 (3)	−0.026 (3)
O5	0.0357 (15)	0.072 (2)	0.0557 (19)	−0.0057 (15)	0.0036 (14)	−0.0073 (16)
C16	0.082 (4)	0.075 (4)	0.060 (3)	−0.011 (3)	−0.007 (3)	−0.009 (3)

### Geometric parameters (Å, º)

**Table:**

I1—C3	2.103 (4)	C5—C6	1.383 (6)
I2—C5	2.111 (4)	C8—C9	1.496 (5)
O1—C2	1.348 (5)	C9—C14	1.398 (5)
O2—C8	1.238 (5)	C9—C10	1.407 (5)
O3—C12	1.374 (4)	C10—C11	1.387 (5)
O4—C11	1.377 (5)	C11—C12	1.421 (5)
O4—C15	1.426 (7)	C12—C13	1.388 (5)
O1—H1	0.8200	C13—C14	1.389 (5)
O3—H3	0.85 (5)	C4—H4	0.9300
O5—C16	1.416 (6)	C6—H6	0.9300
O5—H5	0.85 (3)	C7—H7	0.9300
N1—C7	1.282 (5)	C10—H10	0.9300
N1—N2	1.390 (5)	C13—H13	0.9300
N2—C8	1.365 (5)	C14—H14	0.9300
N2—H2	0.91 (4)	C15—H15C	0.9600
C1—C6	1.414 (5)	C15—H15A	0.9600
C1—C7	1.477 (5)	C15—H15B	0.9600
C1—C2	1.413 (6)	C16—H16A	0.9600
C2—C3	1.404 (6)	C16—H16B	0.9600
C3—C4	1.387 (7)	C16—H16C	0.9600
C4—C5	1.395 (6)		
			
C11—O4—C15	117.3 (3)	C10—C11—C12	119.5 (3)
C2—O1—H1	109.00	O4—C11—C10	125.5 (3)
C12—O3—H3	109 (3)	O3—C12—C11	116.7 (3)
C16—O5—H5	109 (3)	C11—C12—C13	119.7 (3)
N2—N1—C7	117.9 (3)	O3—C12—C13	123.6 (3)
N1—N2—C8	118.4 (3)	C12—C13—C14	120.5 (3)
C8—N2—H2	121 (3)	C9—C14—C13	120.6 (3)
N1—N2—H2	120 (3)	C5—C4—H4	120.00
C2—C1—C7	121.6 (3)	C3—C4—H4	120.00
C6—C1—C7	119.0 (3)	C1—C6—H6	120.00
C2—C1—C6	119.3 (3)	C5—C6—H6	120.00
O1—C2—C3	118.9 (4)	C1—C7—H7	120.00
C1—C2—C3	118.9 (4)	N1—C7—H7	120.00
O1—C2—C1	122.2 (4)	C9—C10—H10	120.00
I1—C3—C4	119.9 (3)	C11—C10—H10	120.00
C2—C3—C4	121.0 (4)	C14—C13—H13	120.00
I1—C3—C2	119.1 (3)	C12—C13—H13	120.00
C3—C4—C5	120.0 (4)	C9—C14—H14	120.00
C4—C5—C6	120.3 (4)	C13—C14—H14	120.00
I2—C5—C4	118.5 (3)	O4—C15—H15B	109.00
I2—C5—C6	121.2 (3)	O4—C15—H15C	109.00
C1—C6—C5	120.5 (4)	O4—C15—H15A	109.00
N1—C7—C1	120.0 (4)	H15A—C15—H15C	109.00
N2—C8—C9	116.5 (3)	H15B—C15—H15C	110.00
O2—C8—N2	122.0 (3)	H15A—C15—H15B	109.00
O2—C8—C9	121.5 (3)	O5—C16—H16A	109.00
C8—C9—C14	123.8 (3)	O5—C16—H16B	110.00
C10—C9—C14	119.2 (3)	O5—C16—H16C	109.00
C8—C9—C10	117.0 (3)	H16A—C16—H16B	109.00
C9—C10—C11	120.6 (3)	H16A—C16—H16C	109.00
O4—C11—C12	115.0 (3)	H16B—C16—H16C	110.00
			
C15—O4—C11—C10	−4.7 (6)	C3—C4—C5—C6	−0.5 (7)
C15—O4—C11—C12	176.2 (4)	I2—C5—C6—C1	179.4 (3)
C7—N1—N2—C8	179.1 (4)	C4—C5—C6—C1	−0.4 (7)
N2—N1—C7—C1	177.4 (3)	O2—C8—C9—C10	16.1 (5)
N1—N2—C8—O2	−2.8 (5)	O2—C8—C9—C14	−164.4 (3)
N1—N2—C8—C9	176.1 (3)	N2—C8—C9—C10	−162.8 (3)
C6—C1—C2—O1	−179.1 (4)	N2—C8—C9—C14	16.8 (5)
C6—C1—C2—C3	1.0 (6)	C8—C9—C10—C11	177.6 (3)
C7—C1—C2—O1	0.8 (6)	C14—C9—C10—C11	−2.0 (5)
C7—C1—C2—C3	−179.1 (4)	C8—C9—C14—C13	−178.4 (3)
C2—C1—C6—C5	0.2 (6)	C10—C9—C14—C13	1.2 (5)
C7—C1—C6—C5	−179.7 (4)	C9—C10—C11—O4	−177.3 (3)
C2—C1—C7—N1	−3.9 (6)	C9—C10—C11—C12	1.7 (5)
C6—C1—C7—N1	176.0 (4)	O4—C11—C12—O3	−1.4 (5)
O1—C2—C3—I1	−1.5 (6)	O4—C11—C12—C13	178.5 (3)
O1—C2—C3—C4	178.2 (4)	C10—C11—C12—O3	179.5 (3)
C1—C2—C3—I1	178.5 (3)	C10—C11—C12—C13	−0.7 (5)
C1—C2—C3—C4	−1.9 (7)	O3—C12—C13—C14	179.7 (3)
I1—C3—C4—C5	−178.7 (3)	C11—C12—C13—C14	−0.2 (5)
C2—C3—C4—C5	1.7 (7)	C12—C13—C14—C9	−0.1 (5)
C3—C4—C5—I2	179.7 (3)		

### Hydrogen-bond geometry (Å, º)

**Table:**

*D*—H···*A*	*D*—H	H···*A*	*D*···*A*	*D*—H···*A*
O1—H1···N1	0.82	1.89	2.614 (4)	147
O5—H5···O2	0.85 (3)	1.87 (2)	2.698 (4)	165 (5)
N2—H2···O3^i^	0.91 (4)	2.17 (5)	3.024 (4)	157 (3)
O3—H3···O5^ii^	0.85 (5)	1.80 (4)	2.643 (4)	170 (4)
C14—H14···O3^i^	0.93	2.55	3.442 (5)	162
C16—H16*A*···O1	0.96	2.51	3.267 (7)	135

Symmetry codes: (i) −*x*+3/2, *y*+1/2, *z*; (ii) *x*+1/2, −*y*+1/2, −*z*+1.
